# Worst-Case Energy Efficiency Maximization in a 5G Massive MIMO-NOMA System

**DOI:** 10.3390/s17092139

**Published:** 2017-09-18

**Authors:** Sunil Chinnadurai, Poongundran Selvaprabhu, Yongchae Jeong, Xueqin Jiang, Moon Ho Lee

**Affiliations:** 1 Department of Electronics and Information Engineering, Chonbuk National University, Jeonju 54896, Korea; sunilkcsss@jbnu.ac.kr (S.C.); poongundran@jbnu.ac.kr (P.S.); ycjeong@jbnu.ac.kr (Y.J.); 2 School of Information Science and Technology, Donghua University, Shanghai 201620, China; xqjiang@dhu.edu.cn

**Keywords:** energy efficiency, beamforming, user pairing, power allocation, 5G, worst-case, massive MIMO, NOMA

## Abstract

In this paper, we examine the robust beamforming design to tackle the energy efficiency (EE) maximization problem in a 5G massive multiple-input multiple-output (MIMO)-non-orthogonal multiple access (NOMA) downlink system with imperfect channel state information (CSI) at the base station. A novel joint user pairing and dynamic power allocation (JUPDPA) algorithm is proposed to minimize the inter user interference and also to enhance the fairness between the users. This work assumes imperfect CSI by adding uncertainties to channel matrices with worst-case model, i.e., ellipsoidal uncertainty model (EUM). A fractional non-convex optimization problem is formulated to maximize the EE subject to the transmit power constraints and the minimum rate requirement for the cell edge user. The designed problem is difficult to solve due to its nonlinear fractional objective function. We firstly employ the properties of fractional programming to transform the non-convex problem into its equivalent parametric form. Then, an efficient iterative algorithm is proposed established on the constrained concave-convex procedure (CCCP) that solves and achieves convergence to a stationary point of the above problem. Finally, Dinkelbach’s algorithm is employed to determine the maximum energy efficiency. Comprehensive numerical results illustrate that the proposed scheme attains higher worst-case energy efficiency as compared with the existing NOMA schemes and the conventional orthogonal multiple access (OMA) scheme.

## 1. Introduction

The demand for wireless traffic is expected to increase a thousandfold by 2020 for the 5G wireless networks due to the requirement of high speed data services, low latency, high spectral efficiency, massive connectivity and green communications [1,2]. Several leading techniques such as massive multiple-input multiple-output (MIMO) [3], spatial modulation (SM) [4,5,6], millimeter (mm) wave [7,8] and non-orthogonal multiple access (NOMA) [9,10] have been considered in recent times to meet the above requirements. In particular, NOMA has attained a compelling interest in a 5G wireless communication system due to its gain in spectral efficiency over the orthogonal multiple access (OMA). NOMA superimposes multiple user signals using power domain at the transmitter and employs successive interference cancellation (SIC) at the receiver side to decode the desired transmitted signal. Existing works on the NOMA system mainly concentrated on the design to increase the performance in terms of spectral efficiency (SE) [11,12]. In addition to spectral efficiency, energy efficiency (EE) is becoming a crucial performance metric due to the swift increase of energy utilization in wireless networks. Efficient use of energy can also cut down the operational expenditure since it aggregates a significant chunk of network cost. EE is defined as the amount of information transmitted per joule of energy consumed [13,14]. In addition, combining NOMA together with MIMO can further increase the SE and EE performance in future wireless networks [15,16,17,18,19]. A significant number of researchers has examined the performance of the NOMA scheme with the distinct topics such as user pairing algorithms, power allocation strategies, beamforming and fairness schemes in order to study the feasibility of adopting the NOMA scheme for 5G communications. The beamforming (BF) technique can minimize the inter beam interference (IBI) by allocating a single beamforming vector to a group of users. Thus, the integration of NOMA with multi-user BF (NOMA-BF) can further enhance the system capacity in future wireless systems [20,21].

However, in order to reduce the inter user interference (IUI), the impact of user pairing was examined for a MIMO-NOMA system in [22,23], where the authors exploit the fixed power allocation (FPA) scheme and pair the users with distinctive channel conditions assuming perfect channel state information (CSI) at the base station (BS). Proportional fairness (PF) based user pairing algorithm is proposed in [24,25] to maximize the achievable rate for the single cell downlink NOMA system. A greedy user pairing and iterative power allocation (PA) algorithm was proposed in [26] to maximize the throughput in an orthogonal frequency division multiplexing (OFDM) based NOMA system. A hierarchical PA scheme is proposed in [27] based on the distinctive channel gains to maximize the sum rate and expedite a bigger number of users in a NOMA system. The authors in [28] proposed a distributed matching algorithm to optimize the user pairing and the transmit power allocation between the near and far users. The proposed user pairing algorithm helps to minimize the inter-user interference (IUI) and maximize the system throughput in a downlink NOMA network. A novel user pairing scheme is proposed in [29] for 5G cellular network, where the users having the largest proportional fairness (PF) coefficient is paired with the users having the best channel conditions in order to mitigate the IUI. A dynamic user scheduling and clustering design is proposed in [30] based on the limited feedback information at the BS to suppress the IUI and inter-cluster interference in the downlink space division multiple access NOMA system. Moreover, the proposed algorithm reduces the system overhead and maximizes the net throughput. The physical layer security of a single-input single-output (SISO) NOMA system was investigated by authors in [31], where they proposed a optimal power allocation strategy to maximize the secrecy sum rate (SSR) and successive interference cancellation (SIC) technique is employed at the receiver side to cancel the IUI. In [32], the authors proposed a low complexity user pairing scheme based on the greedy search method to maximize the weighted sum-rate (WSR) in a downlink NOMA system. A virtual user pairing scheme is proposed in [33], where the multiple near users having a similar channel gain is paired with a single far user to effectively utilize the spectrum in the NOMA system.

We examine the EE maximization problem for multicell massive MIMO-NOMA downlink system with imperfect channel state information at the transmitter (CSIT). Here, we especially consider an ellipsoidal uncertainty model (EUM) [27] to form the CSI errors, where the robust design of the beamforming matrix is taken into account to solve the worst-case EE maximization problem. Since NOMA is an interference bound scheme, we also propose a novel joint user pairing and dynamic power allocation (JUPDPA) algorithm, which mitigates the inter user interference (IUI) and also helps to attain the maximum EE. Several approaches for the EE maximization problem have been proposed in the literature [34,35,36,37] for the perfect CSI at the BSs. For instance, authors in [34] proposed an energy efficient optimal PA technique for a SISO-NOMA system that maximizes the EE subject to the minimum data rate requirement for each user. EE resource allocation problem has been studied in [35], where they proposed a sub optimal resource allocation algorithm to optimize the sub channel and PA factor in order to maximize the EE for a single cell NOMA system. In [36], the authors investigated the EE-SE co-design for the single cell and multi-cell NOMA system considering the perfect CSI at the BS. Successive convex approximation (SCA) based approach is proposed in [37] to tackle the EE maximization problem for a cognitive radio inspired multiuser downlink MISO-NOMA system, which maximizes the EE with respect to the quality of service (QOS) constraint for each primary user. However, in practice, it is generally hard to achieve the perfect CSI in fading channels. Thus, it is meaningful to deal with the imperfect CSI in the problem formulation.

Few related works in [38,39] studied the EE maximization problem considering the imperfect CSI at the transmitter. In particular, authors in [38] examined the EE maximization problem for a MIMO-NOMA system with statistical CSI at the transmitter side and proposed a near optimal PA scheme to maximize the EE. An EE resource allocation algorithm was proposed in [39] to maximize the secret EE and secret key EE for both MISO-single antenna eavesdropper and MIMO-multiple antennas eavesdropper systems considering perfect and statistical CSI at the base station. Although there exists an enormous interest in EE maximization problem, most of the authors considered the problem with the assumption of perfect CSIT or statistical CSIT. Thus, it largely remains unsolved in typical scenarios of interest. For instance, a worst-case beamforming design to maximize the EE for multicell massive MIMO-NOMA system with imperfect CSIT has not been examined before. Motivated from the above observations, the major contribution of this work is summarized as follows.

### 1.1. Summary of Contribution

- In this paper, we investigate the EE maximization problem in a multicell massive MIMO-NOMA downlink system. The EE maximization problem is formulated under the constraints of transmission power and the minimum signal-to-interference-plus-noise-power-ratio (SINR) requirement for the CE users. The considered problem has non-convex fractional objective function, which is intractable and difficult to solve.
- We first turn the fractional objective function into an equivalent parametric one by introducing a non-negative parameter. Successive convex approximation (SCA) method is further employed to solve the dual problem for minimizing the transmission power at the BS and also to find the upper bound values for the original convex optimization problem.
- We propose a joint robust beamforming and EE maximization (JRBEE) algorithm established on the constrained concave–convex procedure (CCCP) and the Dinkelbach method that solve and achieve the convergence to a stationary point of the above problem. The JRBEE algorithm determines the optimal beamforming vector, which helps to maximize the worst-case EE.
- Moreover, we also propose a novel JUPDPA algorithm based on the median and the Euclidean norm of the channel vectors. The JUPDPA algorithm helps to minimize the inter-user interference and also enhance the fairness between the users by dynamically allocating the transmission power to the paired users.
- Simulation results guarantee the convergence property of our proposed EE maximization algorithm and showed that the proposed scheme can significantly improve the worst-case energy efficiency compared to the existing NOMA schemes and the conventional OMA scheme.

### 1.2. Organization

The remainder of this paper is organized as follows. Section 2 describes the considered system model and formulates the worst-case EE maximization problem. A novel JUPDPA scheme is presented in Section 3. A robust beamforming algorithm using CCCP is proposed to tackle the EE maximization problem and find the optimal EE in Section 4. Comprehensive numerical results are presented to show the excellent performance of our proposed scheme in Section 5, and Section 6 concludes this paper.

## 2. System Model and Problem Formulation

**Figure 1:**
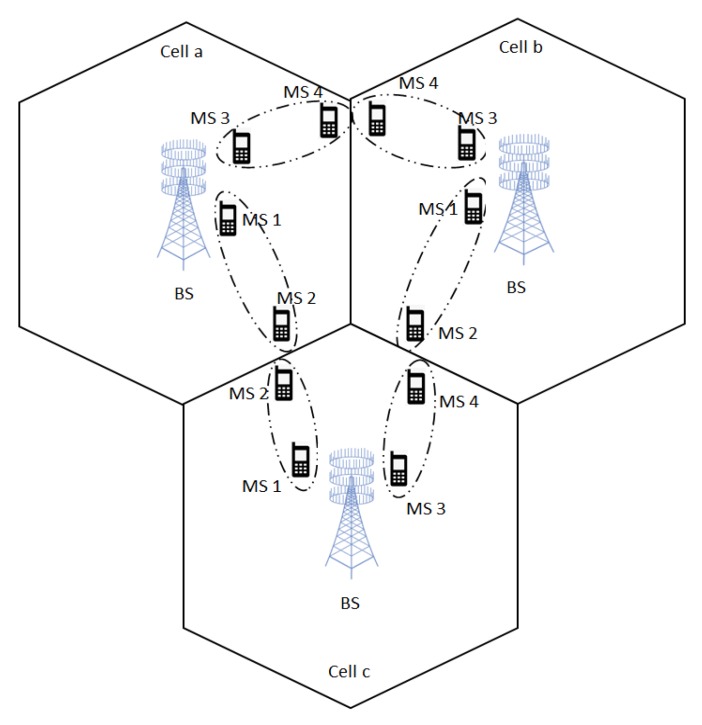
Multicell Massive multiple-input multiple-output non-orthogonal multiple access (MIMO-NOMA) downlink system.

We examine the downlink transmission in a multicell massive MIMO-NOMA system as shown in Figure 1. The considered system model contains N cells, $N_{M}$ users, $N_{B}$ BSs and M pairs. In each cell, the paired users are serviced by multiple antenna BS with $n_{B}>>N_{M}$, where $n_{B}$ and $N_{M}$ is the number of antennas at the BS and number of users present in a single cell, respectively. To expedite the channel estimation at the BS, the users present in each cell simultaneously transmit the training sequences of length $\Phi_{m}$ symbols for every coherence interval *E*, where $N_{M}\leq \Phi_{m}\leq E$.

### 2.1. Transmitter

The transmitted signal vector *x* from $n_{B}$ antennas is given as x=VF, where $V=[V_{1,n},V_{2,n},V_{3,n},\ldots ,V_{M,n}]$ and $F=F_{1,n},F_{2,n},F_{3,n},\ldots ,F_{M,n}$ are the beamforming matrix and the encoded signal for all the *M* pairs, respectively. The BS transmits the symbol vector $T_{1,m,n}$ and $T_{2,m,n}$ in the same time and frequency slot via power domain NOMA. The superimposed signal of the cell center (CC) and cell edge (CE) users belonging to the *m*th pair in *n*th cell is written as

(1) $$\begin{array}{c} F_{m,n}=\sqrt{P_{1,m,n}}T_{1,m,n}+\sqrt{P_{2,m,n}}T_{2,m,n}, \end{array}$$

where $T_{1,m,n}$ and $T_{2,m,n}$ are the desired signal for the CC and CE user, respectively. $P_{1,m,n}$ and $P_{2,m,n}$ are the PA coefficients for the CC and CE user, respectively, which have to satisfy $P_{1,m,n}+P_{2,m,n}=1$ for each user pair. $V_{m,n}\in C^{1\times n_{M,m}}$ represents the beamforming vector corresponding to pair *m*, where m∈M. The transmit power constraints for each BS is given by

(2) $$\begin{array}{c} tr\left\{E\left(x_{p}x_{p}^{H}\right)\right\}\leq P_{p}, \end{array}$$

where $P_{p}$ is the maximum power transmitted at the BS and $p\in N_{B}$.

### 2.2. Channel Model

The overall channel matrix for all the user pairs can be expressed as follows:

(3) $$\begin{array}{c} H=[H_{1,n},H_{2,n},H_{3,n},\ldots ,H_{M,n}], \end{array}$$

where $H\in C^{n_{M}\times n_{B}}$ and *M* is the total number of user pairs in a single cell. The channel matrix corresponding to $m^{th}$ user pair in $n^{th}$ cell is written as

(4) $$\begin{array}{c} H_{m,n}=\;{\hat{H}}_{m,n}+\Psi_{m,n}, \end{array}$$

where $\Psi_{m,n}$ in Equation (4) represents the CSIT uncertainty, which is considered to lie in the bounded ellipsoidal region as

(5) $$\begin{array}{c} H_{m,n}=\left\{{\hat{H}}_{m,n}+\Psi_{m,n}\;|tr\left(\;\Psi_{m,n}G\Psi_{m,n}^{†}\right)\leq \varepsilon^{2}\right\},\forall \;m,n \end{array}$$

where m∈M, n∈N and G⪰0 is a given matrix which represents the shape of the region and $\varepsilon^{2}$ manages the size of the ellipsoidal region. In this paper, we consider that G is of full rank so that $H_{m,n}$ has a structural definition of being an ellipsoid [27]. ${\hat{H}}_{m,n}$ is the minimum mean square error (MMSE) estimate of channel matrix which is obtained from [40] as

(6) $$\begin{array}{c} {\hat{H}}_{m,n}=\frac{\phi_{m,n}p_{m,n}}{\phi_{m,n}p_{m,n}+1}H_{m,n}+\frac{\sqrt{\phi_{m,n}p_{m,n}}}{\phi_{m,n}p_{m,n}+1}B_{m,n}, \end{array}$$

where $p_{m,n}$ is the average transmit power of each uplink pilot symbol and $B_{m,n}$ is a Gaussian matrix with i.i.d. CN (0,1) entries. The users present in each cell simultaneously transmit training sequences of length $\phi_{m,n}$ symbols at the start of every coherence interval.

### 2.3. Receiver

The received signal vector at the *m*th pair in *n*th cell is given as

(7) $$\begin{array}{c} y_{m,n}=y_{1,m,n}+y_{2,m,n}, \end{array}$$

where $y_{1,m,n}$ and $y_{2,m,n}$ are the received signal by CC and CE users, respectively.

#### 2.3.1. Cell Center User

The received signal by the CC user is given as

(8) $$\begin{array}{cc} y_{1,m,n} & =\;h_{1,m,n}^{T}V_{m,n}\sqrt{P_{1,m,n}}T_{1,m,n}+\sum_{j=1,j\neq m}^{M}h_{1,m,n}^{T}V_{j,n}F_{j,n}+z_{1,m,n}, \end{array}$$

where $F_{j,n}=\sqrt{P_{1,j,n}}T_{1,j,n}+\sqrt{P_{2,j,n}}T_{2,j,n}$ and $z_{1,m,n}$ is the additive white gaussian noise (AWGN) noise with $\mathrm{CN}\;(0,\sigma_{1,m,n}^{2})$. In Equation (8), the first term represents the desired signal to a CC user and the second term is due to the interference from other pairs present in the same cell, which we call the inter pair interference (IPI). Inter user interference (IUI) that occurs due to CE users present in the same pair is cancelled due to the implementation of SIC at the CC user. The SINR corresponding to the CC user is represented as

(9) $$\begin{array}{c} \Gamma_{1,m,n}=\frac{P_{1,m,n}|h_{1,m,n}^{T}V_{m,n}{|}^{2}}{\sum_{j=1,j\neq m}^{M}|h_{1,m,n}^{T}V_{j,n}{|}^{2}+\sigma_{1,m,n}^{2}}. \end{array}$$

#### 2.3.2. Cell Edge User

The received signal by the CE user is given as

(10) $$\begin{array}{cc} y_{2,m,n} & =\;h_{2,m,n}^{T}V_{m,n}\sqrt{P_{2,m,n}}T_{2,m,n}+\Xi +\Pi +\sum_{c\in N\backslash n}\sum_{m=1}^{M}g_{2,m,c}^{T}V_{m,c}F_{m,c}+z_{2,m,n}, \end{array}$$

where $\Xi =h_{2,m,n}^{T}V_{m,n}\sqrt{P_{1,m,n}}T_{1,m,n}$, $\Pi =\sum_{j=1,j\neq m}^{M}h_{2,m,n}^{T}V_{j,n}F_{j,n}$ and $z_{2,m,n}$ is the AWGN noise vector with $\mathrm{CN}\;(0,\sigma_{2,m,n}^{2})$. Variable $g_{2,m,c}^{T}$ represents the interfering channel vector (from other cell) to the CE user present at the *m*th pair of *n*th cell. In Equation (10), the first term is the desired signal to CE user and the second term is the interference from CC user present in the same pair which we call it as IUI. The third term is the IPI and the fourth term is due to the interference from the CE users present in other cells, which is called inter cell interference (ICI). The SINR corresponding to the CE user is represented as

(11) $$\begin{array}{c} \Gamma_{2,m,n}=\frac{P_{2,m,n}|h_{2,m,n}^{T}V_{m,n}{|}^{2}}{P_{1,m,n}|h_{1,m,n}^{T}V_{j,n}{|}^{2}+\sum_{j=1,j\neq m}^{M}|h_{2,m,n}^{T}V_{m,n}{|}^{2}+\sum_{c\in N\backslash n}\sum_{m=1}^{M}|g_{2,m,c}^{T}V_{m,c}{|}^{2}+\sigma_{2,m,n}^{2}}. \end{array}$$

#### 2.3.3. Energy Efficiency

The achievable downlink rate of the transmission from BS to $m_{th}$ pair is the mutual information between the unknown transmitted signal $F_{m,n}$ and the observed received signal $y_{m,n},$ and is obtained as

(12) $$\begin{array}{c} R_{m,n}=I\left(F_{m,n};y_{m,n}\right)=\left(R_{1,m,n}+R_{2,m,n}\right), \end{array}$$

where $R_{1,m,n}$ and $R_{2,m,n}$ are the achievable downlink rates by the CC and CE users, respectively. The achievable rates for CC and CE users are given in Equations (13) and (14), respectively, as

(13) $$\begin{array}{cc} R_{1,m,n}= & \;\log_{2}\left(1+\frac{P_{1,m,n}{\left|h_{1,m,n}^{T}V_{m,n}\right|}^{2}}{\sum_{j=1,j\neq m}^{M}{\left|h_{1,m,n}^{T}V_{j,n}\right|}^{2}+\sigma_{1,m,n}^{2}}\right), \end{array}$$

(14) $$\begin{array}{c} R_{2,m,n}=\;\log_{2}\left(1+\frac{P_{2,m,n}{\left|h_{2,m,n}^{T}V_{m,n}\right|}^{2}}{P_{1,m,n}{\left|h_{2,m,n}^{T}V_{m,n}\right|}^{2}+\sum_{j=1,j\neq m}^{M}{\left|h_{2,m,n}^{T}V_{j,n}\right|}^{2}+\sum_{c\in N\backslash n}\sum_{m=1}^{M}{\left|g_{2,m,c}^{T}V_{m,c}\right|}^{2}+\sigma_{2,m,n}^{2}}\right). \end{array}$$

The sum-rate for all pairs is expressed as

(15) $$\begin{array}{c} R_{sum}=\sum_{n\in N}\sum_{m\in M}R_{m,n}, \end{array}$$

where $R_{1,m,n}$ and $R_{2,m,n}$ as given in Equations (13) and (14), respectively. The total transmit power at the base station is given as

(16) $$\begin{array}{c} P_{T}=\sum_{p=1}^{N_{B}}P_{p}+P_{c}, \end{array}$$

where $P_{p}$ is the maximum transmission power at the BS and $P_{c}$ is the circuit power consumption at each BS. Therefore, the energy efficiency (EE) can be defined as the ratio of overall sum rate to the total power consumption, which is expressed as

(17) $$\begin{array}{c} EE=\frac{R_{sum}}{P_{T}}. \end{array}$$

### 2.4. Problem Formulation

The energy efficiency maximization problem for massive MIMO-NOMA system with ellipsoidal uncertainty model (EUM) is formulated as follows

(18) $$\begin{array}{cc} \max_{V\geq 0} & \;\;\min_{H_{m,n}\in H_{m,n}}\;\;EE\\ s.t. & \;\;\;\;\;\;\Gamma_{2,m,n}\geq \Gamma_{2,m,n}^{thd}\\ & \;tr\left(VV^{†}\right)\leq P_{p}, \end{array}$$

where EE is defined in Equation (17) and $\Gamma_{2,m,n}^{thd}$ is the SINR threshold for the CE users. The objective function in (18) maximizes the energy efficiency with bounded channel uncertainties. The constraints given in (18) represent the minimum SINR requirement for the CE users and the transmit power constraint for each BS.

## 3. A Novel Joint User Pairing and Dynamic Power Allocation Algorithm

### 3.1. User Pairing Scheme

In this section, we propose a novel user pairing scheme that reduces the inter-user interference (IUI) between the paired users and also enhance the fairness between them. Algorithm 1 summarizes the steps of the proposed joint user pairing and dynamic power allocation (JUPDPA) algorithm. In Step 1, users are ordered in the descending order based on their Euclidean norm of the channel vector $∥{\hat{h}}_{k,m,n}∥$ and then we split the available users into two categories, i.e., CC and CE with the help of the median given in (19) for the effective user pairing. We define the estimated channel vector from here on as as ${\hat{h}}_{x}$ instead of ${\hat{h}}_{x,m,n}^{T}$ for simplicity. The median (MED) for the total available users in a cell can be expressed as

(19) $$\begin{array}{c} MED=\frac{∥{\hat{h}}_{\frac{N_{M}}{2}}∥+∥{\hat{h}}_{\frac{N_{M}}{2}+1}∥}{2}. \end{array}$$

After splitting, we extricate the users present in both CC and CE group as the odd and even numbered users. The proposed user pairing scheme is given in Step 2, where the odd users from the CC group select the least difference Euclidean norm user from the CE group and even users from the CC group choose the CE user, which has the large difference in the Euclidean norm between them. In Step 3, we eliminate the paired users in Step 2 from the CC and CE user sets and repeat the above steps until CC or CE user set is empty. The transmission power for the paired users will be allocated based on the proposed dynamic power allocation (DPA) scheme in Step 4, while the unpaired users obtain their desired signal power with the help of the conventional OMA scheme in Step 5. Algorithm halts in the final step.

### 3.2. Dynamic Power Allocation Scheme

Once the user pairing is done, we need to allocate the PA coefficients to the CC and CE user. PA is done dynamically for each selected user pair in order to further maximize the EE of the MIMO-NOMA system within each user pair. Before doing that, the PA limit for the CC user needs to be fixed and the difference between the PA for each CC user has to be calculated, which is given as

(20) $$\begin{array}{c} L=\frac{Maximum\;PA\;limit\;for\;CC\;user}{Total\;number\;of\;CC\;users}. \end{array}$$

The PA coefficient for the user closest to the BS in the CC group starts from *L* (derived in (24)) and it will allocate 2L to the next nearest user. This process goes on until the maximum PA limit for the CC user is reached. Once we set the PA coefficient for all the CC users, the power allotted to CE user can be found from $P_{2,m,n}=1-P_{1,m,n}$. The main advantage of the proposed JUPDPA scheme is that the fairness between the CC and CE users are considerably improved due to the increase in the achievable rate of the CE users. In particular, the achievable rate of CE users paired with odd CC user will get the huge benefits from our proposed scheme. Although the achievable rate of few CE users paired with even CC users are slightly degraded due to their least difference Euclidean norm pairing, but it is acceptable considering the improvement in the fairness between the users and also the overall system performance. This enhancement is mainly due to the maximum PA limit for the CC users. In general, energy-efficient transmission strategy transmits data at a very low rate in order to reduce the power consumption. SINR for the CC users is relatively high due to their presence close to the base station (BS) and therefore less transmission power is sufficient to satisfy their data rate requirements. Thus, in our problem, we consider the SINR constraint only on the CE users, which are present far way from the BS and also suffer from low SINR due to the IUI as well as the inter-cell interference (ICI) from the neighboring cells. The proposed JUPDPA algorithm pairs a CC user with the CE user to reduce the IUI. The proposed JUPDPA algorithm also allocates more than 2/3 of the overall transmission power at the BS to CE users in order to meet their data rate requirements and also to improve the system fairness. Moreover, the proposed JRBEE maximization algorithm in Section 4 also helps to attain the data rate requirement for the CE users by finding the optimal beamformers for each pair (CC and CE user), which, in turn, minimize the inter pair interference (IPI) and the power consumption at the BS. The transmit power constraint given in the original problem (18) for each BS helps to mitigate the interference from the neighboring cells to the CE users. It is good to note that the worst-case EE is set to zero, if the maximum transmission power is not large enough to meet the SINR threshold value for the CE users.

**Algorithm 1** Proposed JUPDPA Algorithm.Step1. Initializationandsplitting: Initialize i=1, m=1, $D=\{∥{\hat{h}}_{1}∥>∥{\hat{h}}_{2}∥>,\ldots ,>∥{\hat{h}}_{N_{M}}∥\}$, I={1,2,3,…,I} and J={1,2,3,…,J}.
$$\begin{array}{c} D=\left\{\begin{array}{cc} CC, & \mathrm{if}\;k\leq \;MED,\\ CE, & \mathrm{if}\;k>\;MED, \end{array}\right. \end{array}$$
where *D* represents the Euclidean norm of channel vectors for all the available users in the descending order and $k\in N_{M}$. The users in *D* are categorized into CC and CE group as given in (20) and (21), respectively, based on the above condition and the median (MED) given in (19):(21)$$\begin{array}{cc} CC\;= & \;\;\{∥{\hat{h}}_{1}∥,∥{\hat{h}}_{2}∥,\ldots ,∥{\hat{h}}_{I}∥\}, \end{array}$$
(22)$$\begin{array}{cc} CE\;= & \;\;\{∥{\hat{h}}_{1}∥,∥{\hat{h}}_{2}∥,\ldots ,∥{\hat{h}}_{J}∥\}, \end{array}$$
where *i* and *j* represents the CC and CE user index, respectively, (i∈I and j∈J).Step2. UserPairing: Select a user from CC group and calculate the difference in the Euclidean norm between that user and all the available users from the CE group, which are ordered in descending order. The proposed user pairing scheme is given in the below figure. 
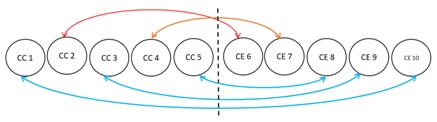
 If the selected user from the CC group is odd numbered, then choose the least difference Euclidean norm user from the CE group for pairing. However, for the even numbered CC user, choose the user from the CE group that has the largest difference in the Euclidean norm of the channel vectors.Step3. RemoveandRepeat: Remove the selected users $H_{m,n}=\left[∥{\hat{h}}_{i}∥,∥{\hat{h}}_{j}∥\right]$ from (20) and (21). Save them in $H=[H_{1,n},H_{2,n},\ldots ,H_{M,n}]$, where *m* represents the user pair index:(23)$$\begin{array}{cc} CC\;= & \;\;CC-\;∥{\hat{h}}_{i}∥,\\ CE\;= & \;\;CE-\;∥{\hat{h}}_{j}∥, \end{array}$$
(24)$$\begin{array}{c} \;\mathrm{Case}:\;\;CC\neq \{\}\;and\;CE\neq \{\}:\\ \;\mathrm{if}\;i<I,\\ i\leftarrow i+1;\;\;m\leftarrow m+1;\\ \mathrm{repeat}\;\;\mathrm{Step}\;\;2.\\ \;\mathrm{else}\\ \mathrm{go}\;\mathrm{to}\;\mathrm{step}\;4. \end{array}$$Step4. PowerAllocation: Paired users will be allocated transmission power based on the dynamic power allocation scheme as given in Table 1.Step5. UnpairedUsers: Remaining unpaired users will be serviced based on the OMA scheme.Step6. Exit: Halt the Algorithm.


Table 1 represents the dynamic PA scheme assuming five CC users, while it is good to note that the proposed scheme can be generalized for any number of CC users following a similar procedure. Based on the above observations, the power allotted to the nearest CC user and farthest CE will be 0.06 and 0.94, respectively, considering five users in the CC group. The overall PA for all the CC users (considered in Algorithm 1) are given in Table 1.

## 4. Proposed Joint Robust Beamforming and Energy Efficiency Maximization Algorithm

Here, in this section, we propose an efficient iterative algorithm based on the CCCP [41,42] and the Dinkelbach algorithm to find the optimal beamforming vector $\left(V_{m,n}^{*}\right)$ for each paired user and determine the maximum energy efficiency, respectively. The Dinkelbach algorithm converts the fractional non-convex optimization problem into a sequence of auxiliary problems while ensuring a super linear convergence rate with limited complexity [43,44]. The worst-case EE maximization problem formulated in (18) is intractable and non-deterministic polynomial hard (NP-hard). Moreover, it is very difficult to solve due to its nonlinear fractional objective function and semi infinite non-convex SINR constraint of the CE users given in (18). In order to solve the above NP-hard problem, we first implement the Dinkelbach algorithm to convert the fractional objective function into a parametric equivalent one with the help of the non-negative parameter λ. The original problem given in (18) is reformulated as follows:

(25) $$\begin{array}{cc} \max_{V} & \min_{H_{m,n}\in H_{m,n}}\;EE(\lambda ,V)\\ s.t. & \;\;\;\;\;\;\Gamma_{2,m,n}\geq \Gamma_{2,m,n}^{thd}\\ & \;tr\left(VV^{†}\right)\leq P_{p}, \end{array}$$

where

(26) $$\begin{array}{c} EE\left(\lambda ,V\right)=R_{sum}\left(V\right)-\lambda P_{T}. \end{array}$$

The objective function of the reformulated problem is still nonlinear, since it is neither concave nor convex and it comes under the category of the difference of convex problem. We follow the CCCP procedure to solve the difference of convex programming problem given in (25). CCCP mainly helps to convert the non-convex problem into a sequence of convex problems by iteratively finding a linear approximation of the original non-convex objective function around the current solution and then solve the approximated convex function in each iteration. In addition, CCCP also guarantees convergence to the stationary point of the original convex optimization problem [41,42]. In the process of converting the above problem into a tractable one, we first define the transmit covariance matrices $L_{m,n}≜V_{m,n}V_{m,n}^{†}⪰0$, since the objective function and constraints present in the original problem (18) are linear in the matrix $VV^{†}$. The optimization problem after substituting the change of variables is as follows

(27) $$\begin{array}{c} \max_{{\left\{L_{m,n}⪰0\right\}}_{m=1}^{M}}\;\;\min_{H_{m,n}\in H_{m,n}}\;\;EE\;(\lambda ,L_{m,n})\\ s.t.\;\;\;\;\;\Gamma_{2,m,n}\left({\left\{L_{m,n}\right\}}_{m=1}^{M}\right)\geq \Gamma_{2,m,n}^{thd}\left({\left\{L_{m,n}\right\}}_{m=1}^{M}\right)\\ \;\;\;\;tr\left(L_{m,n}\right)\leq P_{p}, \end{array}$$

where $EE\;\left(\lambda ,L_{m,n}\right)=R_{sum}\left({\left\{L_{m,n}\right\}}_{m=1}^{M}\right)-\lambda \;P_{T}$ and the achievable rate for CC user is written as

(28) $$\begin{array}{c} R_{1,m,n}\left({\left\{L_{m,n}\right\}}_{m=1}^{M}\right)=f_{1}\left(L_{m,n}\right)+g_{1}\left(L_{m,n}\right), \end{array}$$

where $f_{1}\left(L_{m,n}\right)$ is a concave function and $g_{1}\left(L_{m,n}\right)$ is a convex function, which are given below in (29) and (30), respectively, as

(29) $$\begin{array}{c} f_{1}\left(L_{m,n}\right)=\log_{2}\left(P_{1,m,n}{\left|h_{1,m,n}^{T}V_{m,n}\right|}^{2}+\sum_{j=1,j\neq m}^{M}{\left|h_{1,m,n}^{T}V_{j,n}\right|}^{2}+\sigma_{1,m,n}^{2}\right), \end{array}$$

(30) $$\begin{array}{c} g_{1}\left(L_{m,n}\right)=-\log_{2}\left(\sum_{j=1,j\neq m}^{M}{\left|h_{1,m,n}^{T}V_{j,n}\right|}^{2}+\sigma_{1,m,n}^{2}\right). \end{array}$$

The achievable rate for the CE user is given as

(31) $$\begin{array}{c} R_{2,m,n}\left({\left\{L_{m,n}\right\}}_{m=1}^{M}\right)=f_{2}\left(L_{m,n}\right)+g_{2}\left(L_{m,n}\right), \end{array}$$

where $f_{2}\left(L_{m,n}\right)$ is a concave function and $g_{2}\left(L_{m,n}\right)$ is a convex function, which are given below in (32) and (33), respectively, as

(32) $$f_{2}\left(L_{m,n}\right)=\;\log_{2}\left(P_{2,m,n}{\left|h_{2,m,n}^{T}V_{m,n}\right|}^{2}+P_{1,m,n}{\left|h_{2,m,n}^{T}V_{m,n}\right|}^{2}+\sum_{j=1,j\neq m}^{M}{\left|h_{2,m,n}^{T}V_{j,n}\right|}^{2}+A+\sigma_{2,m,n}^{2}\right),$$

(33) $$\begin{array}{c} g_{2}\left(L_{m,n}\right)=\;-\log_{2}\left(P_{1,m,n}{\left|h_{2,m,n}^{T}V_{m,n}\right|}^{2}+\sum_{j=1,j\neq m}^{M}{\left|h_{2,m,n}^{T}V_{j,n}\right|}^{2}+\sum_{c\in N\backslash n}\sum_{m=1}^{M}{\left|g_{2,m,c}^{T}V_{m,c}\right|}^{2}+\sigma_{2,m,n}^{2}\right), \end{array}$$

where $A≜\sum_{c\in N\backslash n}\sum_{m=1}^{M}{\left|g_{2,m,c}^{T}V_{m,c}\right|}^{2}$. Its good to observe that we have employed the semi definite relaxation (SDR) method to relax the rank constraint, i.e., $rank\{L_{m,n}\}=1$ with the semi definite matrix $L_{m,n}⪰0$ [45]. The non-convex problem in (27) is well approximated by the convex objective function given below in (35). First order Taylor approximation helps to replace $g_{1}\left(L_{m,n}\right)$ and $g_{2}\left(L_{m,n}\right)$ with their convex majorants by the following inequality that supports due to the concavity of log(x) as

(34) $$\begin{array}{c} Z\left(U,C\right)≜log\;det(C)+\frac{1}{ln}tr(C^{-1}\left(U-C\right)). \end{array}$$

Considering the above changes, the optimization problem in (27) can be reformulated as follows:

(35) $$\begin{array}{cc} \max_{{\left\{G_{m,n}⪰0\right\}}_{m=1}^{M}}\;\;\;\;\;EE\left(\lambda ,L_{m,n}\right) & \\ \min_{H_{m,n}\in H_{m,n}}\frac{P_{2}{\hat{h}}_{2}\left(L_{m,n}\right){\hat{h}}_{2}^{†}}{P_{1}{\hat{h}}_{2}\left(L_{m,n}\right){\hat{h}}_{2}^{†}+\Theta +\Delta +\sigma_{2,m,n}^{2}} & \geq \Gamma_{2,m,n}^{thd}\\ tr\left(L_{m,n}\right) & \leq P_{p}, \end{array}$$

where $\Theta =\sum_{j=1,j\neq m}^{M}{\left|h_{2,m,n}^{T}V_{j,n}\right|}^{2}$ and $\Delta =\sum_{c\in N\backslash n}\sum_{m=1}^{M}{\hat{g}}_{2,m,c}\left(L_{m,c}\right){\hat{g}}_{2,m,c}^{†}$. The problem in (35) is still non-convex due to the presence of infinitely many SINR constraints occurred due to the channel uncertainties. Therefore, we need to convert the semi infinite non-convex SINR constraint of the CE user into the linear convex constraint. Fortunately, in literature, we have a well known S-procedure that gives a linear matrix inequality (LMI) representation for adaptive quadratic constraint over an uncertainty set characterized by quadratic inequalities. Equivalent LMI representation for the constraints in (35) are given by converting those infinite constraints into a finite number of linear constraints with the help of auxiliary variables $\alpha_{m}$ for m∈M. For convenience, we cite the S-procedure in [46] here. Let P and Q be two n×n Hermitian matrices, $r\in C^{n}$ and s∈R. Then, the two following conditions are equivalent: (a) $a^{H}Pa+r^{H}a+a^{H}r+s\geq 0$ for all $a\in C^{n}$ such that $a^{H}Qa\leq 1;$ (b) there exists a d∈R such that d≥0 and

(36) $$\begin{array}{c} \left[\begin{array}{cc} P+dQ & r\\ r^{H} & s-d \end{array}\right]⪰0. \end{array}$$

By straightforward implementation of S-procedure, the constraint given in (35) is transformed as

(37) $$\begin{array}{c} \left[\begin{array}{cc} I_{n_{B}}⊗\Omega_{m,n}+\alpha_{m,n}I_{n_{B}}⊗G_{m,n} & vec({\hat{h}}_{2,m,n}\Omega_{m,n})\\ vec{\left(\Omega_{m,n}{\hat{h}}_{2,m,n}\right)}^{†} & \chi \end{array}\right]⪰0, \end{array}$$

where $\alpha_{m,n}\geq 0$, m={1,2,3,…,M},

(38) $$\begin{array}{c} \chi =tr\left({\hat{h}}_{2,m,n}\Omega_{m,n}{\hat{h}}_{2,m,n}^{†}\right)-\Gamma_{2,m,n}^{thd}I-\alpha_{m,n}\varepsilon^{2}, \end{array}$$

(39) $$\begin{array}{c} \Omega_{m,n}=P_{2,m,n}L_{m,n}-\Gamma_{2,m,n}^{thd}\left(P_{1,m,n}L_{m,n}+\sum_{j=1,j\neq m}^{M}L_{j,n}+B+\sum_{c\in N\backslash n}L_{m,c}+\sigma_{2,m,n}^{2}\right), \end{array}$$

where $B=\sum_{c\in N\backslash n}\sum_{m=1}^{M}{\hat{g}}_{2,m,c}\left(L_{m,c}\right){\hat{g}}_{2,m,c}^{†}$. It is worth mentioning here that the uncertainty present in the transformed constraint is now dealt by the parameters i.e., $G_{m,n}$ and ε. Now, problem (35) can be equivalently reformulated to a convex optimization problem as given below:

(40) $$\max_{{\left\{G_{m,n}⪰0\right\}}_{m=1}^{M}}\;EE\;\left(\lambda ,L_{m,n}\right)\;\begin{array}{cc} s.t.\;\;\;M_{m}\left({\left\{L_{m,n}\right\}}_{m=1}^{M}\right) & ⪰0\\ tr\left(L_{m,n}\right) & \leq P_{p}, \end{array}$$

where

(41) $$\begin{array}{c} M_{m}\left({\left\{L_{m,n}\right\}}_{m=1}^{M}\right)=\left[\begin{array}{cc} I_{n_{B}}⊗\Omega_{m,n}+\alpha_{m,n}I_{n_{B}}⊗G_{m,n} & vec({\hat{h}}_{m,n}\Omega_{m,n})\\ vec{\left(\Omega_{m,n}{\hat{h}}_{m,n}\right)}^{†} & tr\left({\hat{h}}_{2,m,n}\Omega_{m,n}{\hat{h}}_{2,m,n}^{†}\right)-\Gamma_{2,m,n}^{thd}I-\alpha_{m,n}\varepsilon^{2} \end{array}\right]. \end{array}$$

Now, we observe that (40) is a tractable convex problem that can be solved with the help of convex packages available in the literature [47]. However, we can find the sub optimal beamforming vector for each pair via solving the problem in (40), and the total transmit power is increased to maintain the required SINR for each CE user. Thus, in order to enhance the accuracy of first order Taylor approximation and to find the upper bound values for the original convex optimization problem, and we consider the dual problem of power minimization at the base station under the SINR constraint for the CE user. The dual optimization problem is formulated subject to the SINR constraints for all the CE users is as follows:

(42) $$\begin{array}{c} \min_{{\left\{G_{m,n}⪰0\right\}}_{m=1}^{M}}\;\;\sum_{p=1}^{N_{B}}\;\;C_{m}\left({\left\{L_{m,n}\right\}}_{m=1}^{M}\right)\\ \;s.t.\;\;\;\;\min_{\psi_{m}\in H_{m}}\;\frac{P_{2}T_{2,m,n}}{P_{1}T_{1,m,n}+\Theta +\Delta +\sigma_{2,m,n}^{2}}\geq \Gamma_{2,m,n}^{thd}, \end{array}$$

where $T_{1,m,n}={\hat{h}}_{1,m,n}^{†}L_{m,n}{\hat{h}}_{1,m,n}$, $T_{2,m,n}={\hat{h}}_{2,m,n}^{†}L_{m,n}{\hat{h}}_{2,m,n}$ and

(43) $$\begin{array}{c} C_{m}\left({\left\{L_{m,n}\right\}}_{m=1}^{M}\right)=tr\left(\sum_{m=1}^{M}L_{m,n}\right). \end{array}$$

The considered dual problem is non-convex due to the infinite number of SINR constraints on the CE user. To solve the above problem, we employ the successive convex approximation (SCA) approach [47] and further implement S-Lemma to convert the infinite number of SINR constraints into a finite one. The sub-optimal beamforming vectors obtained by solving the convex problem in (40) is considered as the initial values for the transmit power minimization problem. The convex reformulated problem for minimizing the transmission power is given as

(44) $$\begin{array}{c} \min_{{\left\{L_{m,n}⪰0\right\}}_{m=1}^{M}}\;\;\sum_{p=1}^{N_{B}}\;\;C_{m}\left({\left\{L_{m,n}\right\}}_{m=1}^{M}\right)\\ \;s.t.\;\;\;\;\;\;\;\;\;\;\;\;M_{m}\left({\left\{L_{m,n}\right\}}_{m=1}^{M}\right)⪰0, \end{array}$$

where $M_{m}\left({\left\{L_{m,n}\right\}}_{m=1}^{M}\right)$ are already given in (41). Algorithm 2 summarizes the steps of the proposed joint robust beamforming and energy efficiency (JRBEE) maximization algorithm where the optimal beamforming vectors are obtained iteratively to maximize the EE. It is good to note that the non-decreasing optimal values attained over the proposed JRBEE maximization algorithm can minimize the power consumption at the BS. Since both (45) and (46) are convex, updating $L_{m,n}$ iteratively will only increase or maintain the objective value, which converges to a stationary solution of the original problem (35). The optimal beamforming vectors $V_{m,n}^{*}$ found with the proposed JRBEE maximization algorithm are substituted in (17) to maximize the EE by finding the optimal value i.e., $\lambda^{*}$, where it yields $EE(\lambda^{*},V_{m,n}^{*})=0$. In the proposed JRBEE maximization algorithm, we first initialize ${\left\{L_{m,n}^{\left(k\right)}\right\}}_{m=1}^{M}$ to arbitrary positive semidefinite matrix, which is feasible for the problem (27). The initialization value plays a key role in deciding the convergence rate of the proposed JRBEE maximization algorithm. If the chosen arbitrary initialization value is very close to the optimal solution, then it takes only fewer iterations to attain the stationary point. Otherwise, if the chosen arbitrary initial value is not close to the optimal solution, then the proposed iterative algorithm will still achieve the same objective value as the previous one, but the number of iterations taken for the convergence will increase. We then initialize the matrix ${\left\{L_{m,n}^{\left(l\right)}\right\}}_{m=1}^{M}$ for the transmit power minimization problem (46) by using the beamforming vector $\left({\bar{V}}_{m}\right)$ obtained from solving the problem (45) in the proposed JRBEE maximization algorithm. We also initialize $\lambda_{init}=4$ for the outer loop iteration, which employs Dinkelbach method to determine the maximum EE. Moreover, Figure 3 in Section 5 proves that the proposed energy efficient maximization scheme converge to a stationary point within few iterations using the arbitrary feasible initialization point.

**Algorithm 2** Proposed JRBEE maximization Algorithm.1. Initialize: Input: $\lambda_{init}$ and let $\lambda =\lambda_{init}$. Output: $V_{m,n}^{*}$ and $\lambda^{*}$2. Repeat:2.1 Initialize the variable k=1, ${\left\{L_{m,n}^{\left(k\right)}\right\}}_{m=1}^{M}⪰0$ to a arbitrary positive semidefinite matrix (feasible) Repeat
1:**for** k←k+1 **do**2:Update the variable ${\left\{L_{m,n}^{\left(k+1\right)}\right\}}_{m=1}^{M}$ as a solution to the following convex problem
(45)$$\max_{{\left\{L_{m,n}⪰0\right\}}_{m=1}^{M}}\;\;\;{R^{\prime }}_{sum}(\{L_{m,n}^{\left(k+1\right)},L_{m,n}^{\left(k\right)}{\}}_{m=1}^{M})-\lambda \;P_{T}\;\begin{array}{cc} s.t.\;{M^{\prime }}_{m}\left({\left\{L_{m,n}^{\left(k+1\right)}\right\}}_{m=1}^{M}\right) & ⪰0\\ tr\left(L_{m,n}^{\left(k+1\right)}\right) & \leq P_{p}, \end{array}$$
until convergence criterion is satisfied.3:**end for**2.2 Calculate the linear beamformer $\left({\bar{V}}_{m}\right)$ for *m* pairs and SINR for all users.2.3 Initialize ${\left\{L_{m,n}^{\left(l\right)}\right\}}_{m=1}^{M}$ with the help of $\left({\bar{V}}_{m}\right)$ obtained by solving (45) and set l=1.Repeat
1:**for** l←l+1 **do**2:Update the variable ${\left\{L_{m,n}^{\left(l+1\right)}\right\}}_{m=1}^{M}$ as a solution to the following convex problem
(46)$$\begin{array}{c} \min_{{\left\{L_{m}⪰0\right\}}_{m=1}^{M}}\;\;{C^{\prime }}_{m}\left(L_{m,n}^{\left(l+1\right)}\right)\\ \;s.t.\;\;\;\;\;\;{M^{\prime }}_{m}\left(L_{m,n}^{\left(l+1\right)}\right)\geq 0, \end{array}$$
until convergence criterion is satisfied. 3:**end for**2.4 Determine the optimal beamformer $\left(V_{m,n}^{*}\right)$ that achieves minimum transmission power.3. Obtain the EE by substituting $V_{m,n}^{*}$ into the (26) and find the value of λ.4. Update λ in step 1 and repeat the procedure until the convergence criteria is satisfied to find $\lambda^{*}$.

In Algorithm 2

(47) $$\begin{array}{c} {R^{\prime }}_{sum}\left({\left\{L_{m,n}^{\left(k+1\right)},L_{m,n}^{\left(k\right)}\right\}}_{m=1}^{M}\right)=R_{1,m,n}^{\prime }\left({\left\{L_{m,n}^{\left(k+1\right)},L_{m,n}^{\left(k\right)}\right\}}_{m=1}^{M}\right)+R_{2,m,n}^{\prime }\left({\left\{L_{m,n}^{\left(k+1\right)},L_{m,n}^{\left(k\right)}\right\}}_{m=1}^{M}\right), \end{array}$$

(48) $$\begin{array}{c} {M^{\prime }}_{m}\left(L_{m,n}^{\left(D+1\right)}\right)=\left[\begin{array}{cc} I_{n_{B}}⊗\Omega_{m,n}+\alpha_{m,n}I_{n_{B}}⊗G_{m,n} & vec({\hat{h}}_{m,n}\Omega_{m,n})\\ vec{\left(\Omega_{m,n}{\hat{h}}_{m,n}\right)}^{†} & tr\left({\hat{h}}_{2,m,n}\Omega_{m,n}{\hat{h}}_{2,m,n}^{†}\right)-\Gamma_{2,m,n}^{thd}I-\alpha_{m,n}\varepsilon^{2} \end{array}\right], \end{array}$$

(49) $$\begin{array}{c} \Omega_{m,n}=P_{2,m,n}L_{m,n}^{\left(D+1\right)}-\Gamma_{2,m,n}^{thd}\left(P_{1,m,n}L_{m,n}^{\left(D+1\right)}+\sum_{j=1,j\neq m}^{M}L_{j,n}^{\left(D+1\right)}+B+\sum_{j\in M\backslash m}L_{m,c}^{\left(D+1\right)}+\sigma_{2,m,n}^{2}\right), \end{array}$$

$C_{m}^{\prime }\left({\left\{L_{m,n}^{\left(l+1\right)}\right\}}_{m=1}^{M}\right)=tr\left(\sum_{m=1}^{M}L_{m,n}^{\left(l+1\right)}\right)$, $B=\sum_{c\in N\backslash n}\sum_{m=1}^{M}{\hat{g}}_{2,m,c}\left(L_{m,c}^{\left(D+1\right)}\right){\hat{g}}_{2,m,c}^{†}$ and D∈{k,l}.

### Complexity Analysis for the Proposed Algorithms

We first calculate the complexity of the proposed JUPDPA algorithm, where all the available users are split into two categories, i.e., CC and CE users. Each pair contains only two users to avoid the SIC complexity at the receiver side. CC users chose their corresponding paired user based on the proposed JUPDPA algorithm by exhaustively searching all the available users in the CE category. Thus, the computational complexity of the proposed JUPDPA algorithm is $O(\frac{\frac{N_{M}}{2}!}{\Pi_{m=1}^{M}J_{m}!})$, where $N_{M}$ and $J_{m}$ is the total number of available users and the number of users present in each pair (we assume two users per pair), respectively. The computation complexity of the proposed JRBEE maximization algorithm mainly consists of two parts. The first part is the required number of iterations taken for the convergence and the second part is the complexity taken to solve the convex optimization problem in (45) and (46) during each iteration. The overall computational complexity for the proposed JRBEE maximization algorithm is $O(Z_{3}\left(\left(Z_{1}+Z_{2}\right){\left(N_{M}N_{B}\right)}^{3.5}log\mu^{-1}\right))$, where $Z_{1}$ and $Z_{2}$ are the two inner loop iterations required to solve the convex problem (45) and (46), respectively. $Z_{3}$ is the number of outer loop iterations required to determine the maximum EE using the Dinkelbach method, which is known for its super linear convergence rate. $N_{B}$ and μ is the number of BSs and the solution accuracy, respectively. The convex (CVX) tool is used to solve the above convex optimization problems using interior point approach, which takes $O({\left(N_{M}N_{B}\right)}^{3.5}log\mu^{-1})$ computations [48]. The proposed energy efficiency maximization (EEmax) scheme (JUPDPA + JRBEE maximization algorithm) has the least implementation complexity due to the simpler matrix calculations. Average run-time computed in Figure 4 also proves that the proposed EEmax scheme takes less time to attain the objective value. The beamforming matrix is determined as $V_{m,n}\leftarrow B_{m,n}C_{m,n}^{1/2}$, where the diagonal elements present in $C_{m,n}$ matrix represents the non-zero eigenvalues of $L_{m,n}^{D}$ (D∈{k,l}) and the corresponding eigenvectors are present in the column space of the matrix $B_{m,n}$.

## 5. Numerical Results and Discussion

In this section, we evaluate the performance of our proposed EEmax scheme (includes JUPDPA and JRBEE algorithm) and compare it with the existing NOMA schemes in [20,21] and the conventional OMA scheme [49]. The proposed JUPDPA in Algorithm 1 and JRBEE algorithm given in Algorithm 2 are jointly labeled as proposed EEmax Scheme. The compared existing schemes are as follows: (1) we obtain the EE considering EUM when the sum rate is maximized for the iterative beamforming algorithm proposed in [20] referred to as Ratemax scheme in the sequel; (2) user pairing and PA algorithm along with the zero-forcing beamforming (ZFBF) proposed in [21] is evaluated for EUM to achieve the EE referred to as EE-ZFBF scheme. Moreover, the OMA transmission with imperfect CSI is considered where two users transmit in two orthogonal time slots and the robust beamforming design is utilized to tackle the corresponding optimization problem via CCCP to maximize the EE, labeled as OMA scheme [49]. We consider a multi cell massive MIMO-NOMA downlink system as shown in Figure 1. We assume there are three cells, $N_{B}=3$ base stations, each with multiple transmit antennas and $N_{M}$ users, each equipped with a single receive antenna. Each BS is subject to the same transmit power constraint. Unless otherwise specified, we assume channel uncertainty (error bound) for EUM is ε=0.05, β=0dB, $\Gamma_{2,m,n}^{thd}$ = 10 dB, $N_{M}$ = 10, $n_{B}$ = 20, $\lambda_{init}=4$, $P_{P}$ = 30 dBm, $P_{C}$ = 1 W, and $\sigma_{m,n}^{2}=1$. We will examine the worst-case energy efficiency (EE) performance for EUM. Worst-case EE was achieved by averaging the energy efficiency over the acquisition of fading channel matrices. Unless explicitly stated, all results are presented for 20 channel fading realizations. Jain’s fairness index [50] is employed to measure the fairness between the users. We also presume that the elements of the channel matrices between the user in the $b^{th}$ cell and BS in the $a^{th}$ cell follows independent and identically distributed (i.i.d.) complex Gaussian with $\mathrm{CN}\;\left(0,\beta^{|a-b|}\right)$ distribution in which we call β the inter-cell channel gain. We consider only two users in each pair to reduce the SIC receiver’s decoding complexity. Convergence criteria is set to $10^{-4}$, i.e., we assume that the proposed JRBEE algorithm comes to halt when the difference between attained worst-case EE values within the successive iterations are less than $10^{-4}$.

**Figure 2:**
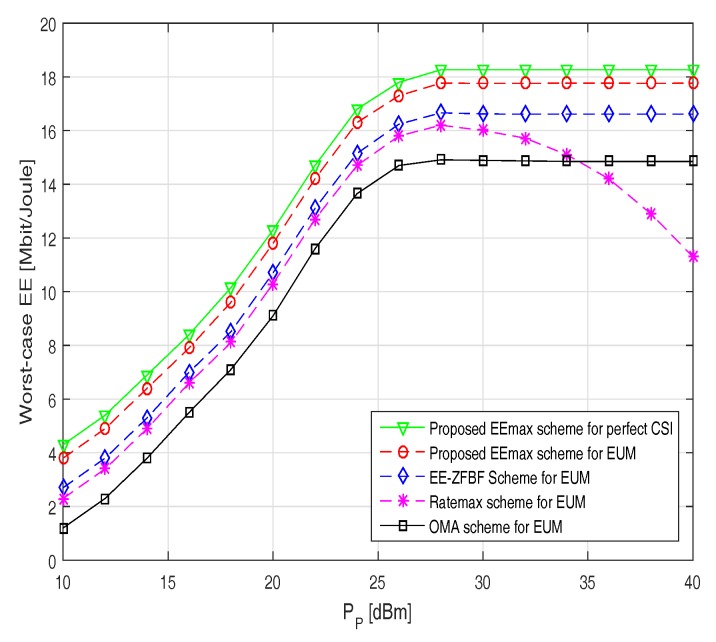
Worst-case energy efficiency (EE) versus transmission power $P_{P}$.

Figure 2 demonstrates the worst-case energy efficiency versus available transmission power at the BS with the fixed circuit power of $P_{C}$ = 1 W. It can be observed that the proposed EEmax scheme consistently outperforms the Ratemax and OMA scheme in terms of the worst-case EE. It can be noticed that the worst-case EE of the proposed scheme increases until the transmission power reaches 28 dBm and begins to saturate after that level. This can be explained considering that the BS stops consuming more power to transmit the signals when the proposed EEmax scheme achieves the optimal EE for the given convergence criteria. In particular at $P_{P}$ = 30 dBm, the worst-case EE achieved by the proposed EEmax scheme is 17.6 Mbits/joule compared to 16.5 Mbits/joule by the EE-ZFBF scheme. The worst-case EE for the Ratemax scheme increases until the power reaches 28 dBm and then it begins to decrease, since the scheme emphasis on finding the BF vectors to achieve the high transmission capacity. It is also good to note that the worst-case EE performance of the proposed EEmax scheme for EUM performs close to the case with perfect CSI.

**Figure 3:**
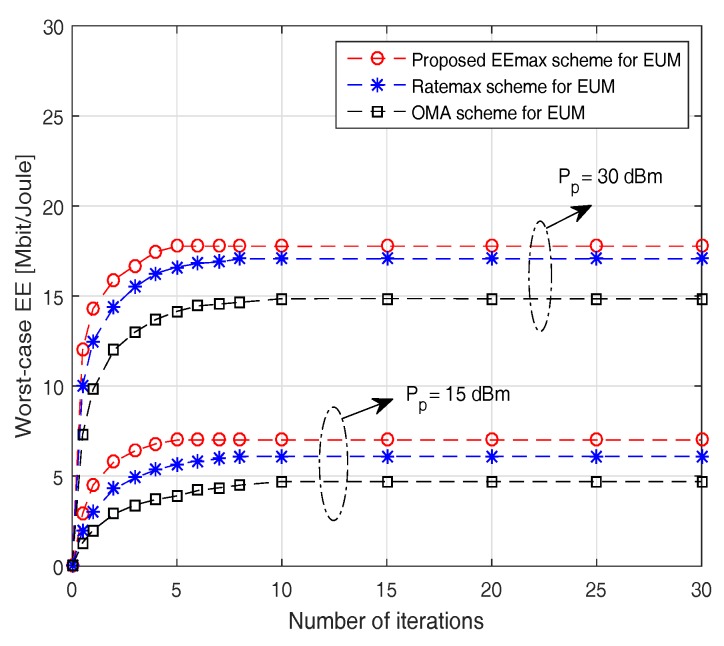
Worst-case EE versus number of iterations.

Figure 3 illustrates the worst-case EE versus number of iterations for two different transmission powers at the BS. It can be noticed that the worst-case EE increases monotonically for the proposed EEmax scheme before it converges at around five iterations for both low ($P_{P}$ = 15 dBm) and high ($P_{P}$ = 30 dBm) transmission power. However, the Ratemax and OMA scheme takes around eight and ten iterations, respectively, for convergence. This is due to the reason that the optimal BF vectors in the proposed JRBEE algorithm are obtained by minimizing the transmission power at the base station. We also note that the proposed EEmax scheme consistently beats the Ratemax scheme in terms of the worst-case EE and the number of iterations to attain convergence.

**Figure 4:**
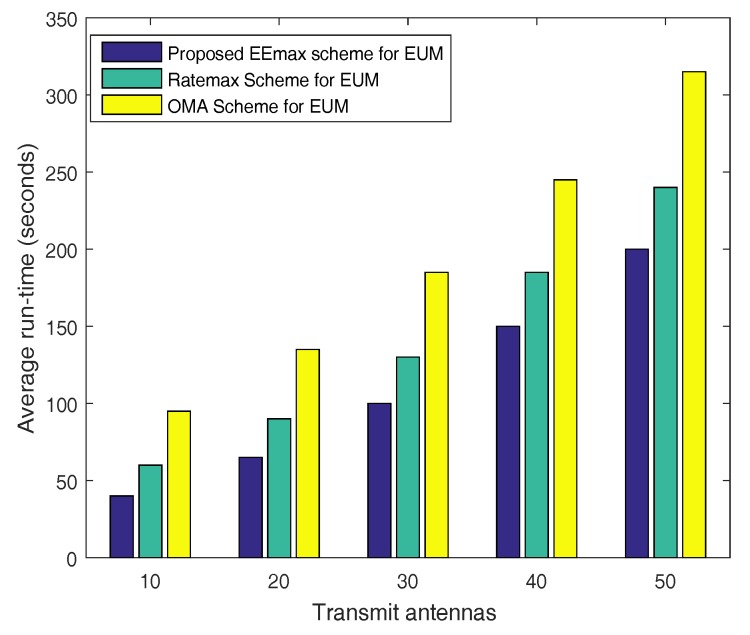
Average run-time versus number of transmit antennas.

Figure 4 depicts the number of antennas at the transmitter versus an average CPU run time, which helps to determine the implementation complexity of the considered schemes. It can be seen that the proposed EEmax scheme has the least implementation complexity compared to the Ratemax and the OMA scheme. In particular, the average runtime taken by the proposed scheme for 20 antennas is only 65 s, whereas the Ratemax scheme and the OMA scheme takes around 90 s and 135 s, respectively. This observation can be justified for the reason that the proposed user pairing algorithm splits the available $N_{M}$ users into two groups and search only $\frac{N_{M}}{2}$ times to allocate the transmission power for selecting one active user pair. Moreover, the proposed EEmax scheme also avoids the large matrix inversions and complex matrix calculations.

**Figure 5:**
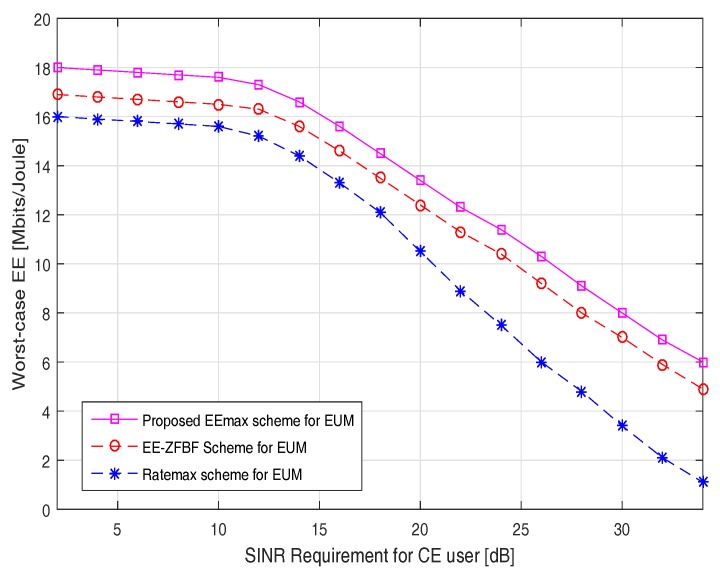
Worst-case EE versus signal-to-interference-plus-noise-power-ratio (SINR) requirement for cell edge (CE) users.

Figure 5 investigates the worst-case EE versus SINR requirement for the CE user. It can be observed that the proposed EEmax scheme consistently outperforms the EE-ZFBF and Ratemax schemes in terms of the worst-case EE. The worst-case EE gradually decreases for all the schemes while there is a increment in the minimum SINR requirement for the CE users. In particular, at SINR = 20 dB, the worst-case EE attained by the proposed EEmax scheme is 13.4 Mbits/joule compared to the 12.5 Mbits/joule and 10.5 Mbits/joule achieved by the EE-ZFBF and the Ratemax scheme, respectively. This performance gain can be validated in considering that the proposed EEmax scheme allocates more than 2/3 of the total available transmission power to the CE users. Moreover, the BF vectors attained by the proposed JRBEE maximization algorithm are robust even in the worst-case conditions. It is also good to note that the worst-case EE is set to zero if the transmit power is not large enough to meet the SINR requirement of the CE users.

**Figure 6:**
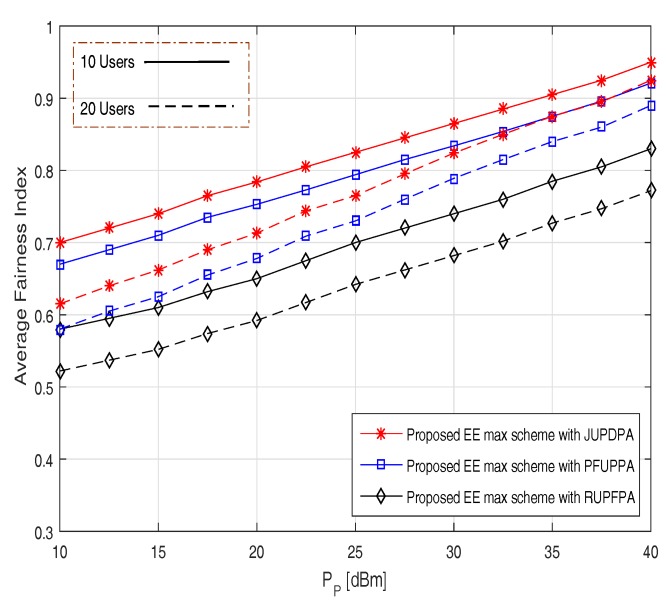
Average Fairness Index versus transmission power $P_{P}$.

Figure 6 exemplifies the average fairness index versus transmission power at the BS for a different number of users. We compare our proposed JUPDPA algorithm with RUPFPA (random user pairing with FPA) [22] and PFUPPA (proportional fairness based user pairing and PA) [23] algorithm. It can be noticed that the fairness linearly increases with the transmission power with the proposed JUPDPA algorithm providing the best fairness among the considered schemes. In particular, while considering for 10 users at $P_{P}$ = 25 dBm, the average fairness index is 0.83 for the proposed JUDPA algorithm compared to 0.79 and 0.7 for the PFUPPA and RUPFPA algorithm, respectively. This was expected because the proposed pairing scheme has a significantly greater impact on the achievable rate of the CE users, which, in turn, helps to increase the fairness between the users. Moreover, it is good to note that the average fairness index is reduced for a large number of users due to increase in the IUI.

**Figure 7:**
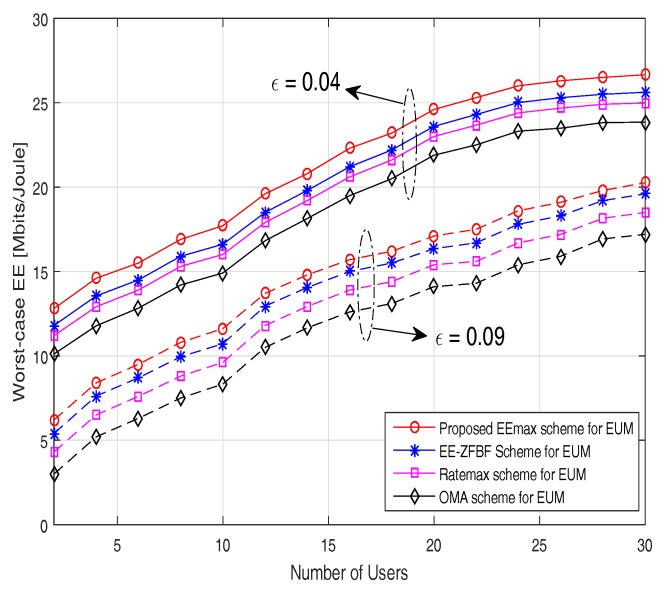
Worst-case EE versus number of users.

In Figure 7, the worst-case energy efficiency is evaluated versus the number of users for different channel uncertainty values. It can be noticed that when the number of users increases, the worst-case energy efficiency also increases only until all the users needs can be satisfied by the total available power at the BS. Once it exceeds that limit, then the worst-case EE will start to decrease. Worst-case EE attained by the proposed EEmax scheme decreases with an increase of channel uncertainty ϵ as expected. Our proposed EEmax scheme again achieves better performance than the other considered schemes. In particular, at ϵ = 0.09 for 10 users, the worst-case energy efficiency for the proposed EEmax scheme is 11.5 Mbits/joule compared to 8.5 Mbits/joule attained by the OMA scheme. This can be justified by the fact that the conventional OMA scheme does not fully utilize the available spectrum and energy resources compared to NOMA scheme where a greater number of users can be served simultaneously by the power domain multiplexing.

## 6. Conclusions

In this paper, a robust beamforming design was studied to solve the EE maximization problem in a 5G massive MIMO-NOMA system with imperfect CSI at the BS. In particular, we have considered the EUM for the inclusion of CSI errors. In order to tackle the above problem, we first converted the fractional nonlinear objective function into its equivalent parametric form. An efficient iterative algorithm based on the CCCP was proposed to optimize the transmit beamforming vectors for each pair. We then employed the Dinkelbach method to find the optimal parameter that maximize the worst-case EE. We also proposed a novel JUPDPA algorithm that improves the fairness between the users and maximize the worst-case EE by dynamically allocating the transmission power to the paired users. Numerical results guarantee that the proposed EEmax scheme converges faster than the Ratemax scheme and the OMA scheme. Via numerical results, it was also confirmed that the worst-case EE achieved by the proposed EEmax scheme persistently outperforms the existing NOMA schemes and the conventional OMA scheme.

## Figures and Tables

**Table 1:** Proposed Dynamic Power Allocation Scheme.

CC Users	CC 1	CC 2	CC 3	CC 4	CC 5
Power Allocation (PA) Coefficients	L (0.06)	2L (0.12)	3L (0.18)	4L (0.24)	5L (0.30)

## Abbreviations

The following abbreviations are used in this manuscript:

JUPDPA	joint user pairing and dynamic power allocation
JRBEE	joint robust beamforming and energy efficiency maximization
EUM	ellipsoidal uncertainty model
EE	energy efficiency
SDR	semi definite relaxation
SCA	successive convex approximation
SINR	signal-to-interference-plus-noise-power-ratio
EEmax	energy efficiency maximization
CCCP	constrained concave-convex procedure
ZFBF	zero forcing beamforming
FPA	fixed power allocation
Ratemax	achievable rate maximization
NOMA	non-orthogonal multiple access
OMA	orthogonal multiple access
MIMO	multiple-input-multiple-output
CSIT	channel state information at the transmitter
CSI	channel state information
BS	base station
i.i.d	independent and identically distributed
MMSE	minimum mean square error
RUPFPA	random user pairing and fixed power allocation
PFUPPA	proportional fairness based user pairing and power allocation
DPA	dynamic power allocation
